# Glycemic Variability and Oxidative Stress: A Link between Diabetes and Cardiovascular Disease?

**DOI:** 10.3390/ijms151018381

**Published:** 2014-10-13

**Authors:** Yoshifumi Saisho

**Affiliations:** Department of Internal Medicine, Keio University School of Medicine, 35 Shinanomachi, Shinjuku-ku, Tokyo 160-8582, Japan; E-Mail: ysaisho@z5.keio.jp; Tel.: +81-3-5363-3797; Fax: +81-3-3359-2745

**Keywords:** glycemic variability, oxidative stress, diabetes, cardiovascular disease, hypoglycemia

## Abstract

Diabetes is associated with a two to three-fold increase in risk of cardiovascular disease. However, intensive glucose-lowering therapy aiming at reducing HbA1c to a near-normal level failed to suppress cardiovascular events in recent randomized controlled trials. HbA1c reflects average glucose level rather than glycemic variability. In *in vivo* and *in vitro* studies, glycemic variability has been shown to be associated with greater reactive oxygen species production and vascular damage, compared to chronic hyperglycemia. These findings suggest that management of glycemic variability may reduce cardiovascular disease in patients with diabetes; however, clinical studies have shown conflicting results. This review summarizes the current knowledge on glycemic variability and oxidative stress, and discusses the clinical implications.

## 1. Introduction

The number of people with diabetes are continuously increasing all over the world. People with diabetes are at two to three-fold increased risk of developing cardiovascular disease (CVD), and CVD remains the major cause of death in patients with diabetes [1,2].

In the Diabetes Control and Complications Trial/Epidemiology of Diabetes Interventions and Complications (DCCT/EDIC) and the UK Prospective Diabetes Study (UKPDS), intensive glycemic control has been shown to reduce the development of CVD as well as diabetic microangiopathy during long-term follow up in patients with both type 1 (T1DM) and type 2 diabetes (T2DM), so-called legacy effects [3,4]. On the other hand, recent clinical trials aiming at reducing HbA1c to a near-normal level in patients with T2DM have failed to show an additional benefit on CVD outcomes [5,6,7]. In the Action to Control Cardiovascular Risk in Diabetes (ACCORD) trial, the trial was stopped because of a significant increase in all-cause mortality in patients with T2DM who were randomized to the intensive glycemic control group [5].

A U-shaped association between HbA1c and CVD or all-cause mortality in patients with diabetes has been also reported by Currie *et al.* [8]. These findings suggest that normalization of HbA1c does not necessarily improve CVD outcomes, and that other factors are associated with these outcomes independent of HbA1c.

Glycemic variability (GV) has been proposed as one of the factors associated with CVD outcomes in patients with and without T2DM. This review summarizes current knowledge of GV and its association with oxidative stress and CVD, and discusses its clinical implication in the treatment of diabetes.

## 2. Assessment of Glycemic Variability

**Figure 1:**
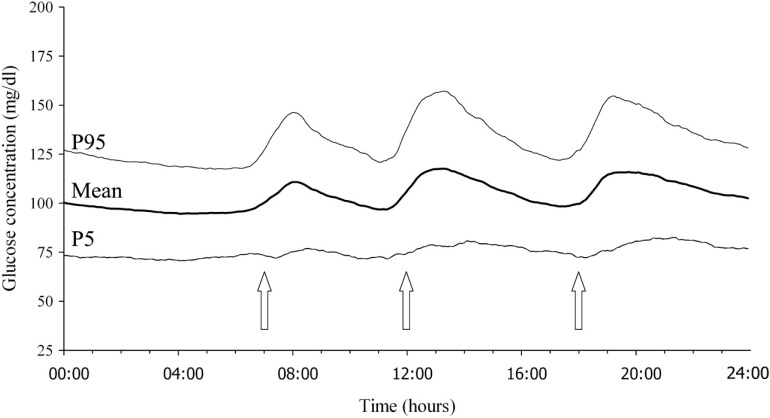
Daily glucose profile in healthy subjects assessed by continuous glucose monitoring. The central line is the mean, and the two outer lines represent the 5th and 95th percentiles (P5 and P95, respectively). Arrows indicate the times of three meals during a day. Reproduced with permission from the American Diabetes Association [9].

Normally, plasma glucose levels are kept within a narrow range, of 80–120 mg/dL, throughout the day in people with normal glucose tolerance (NGT) (Figure 1) [9]. Once glucose intolerance develops, glycemic swings become greater. One of the reasons for confusion on GV is that there are various definitions or concepts of GV. In general, GV refers to intra-day GV or day-to-day GV, but it also may refer to visit-to-visit GV over months to years (Table 1). While glucose values measured by self-monitoring of blood glucose (SMBG) or continuous glucose monitoring (CGM) are usually used to assess intra-day or day-to-day GV, fasting plasma glucose (FPG) and HbA1c are also used to assess visit-to-visit GV. Furthermore, although GV usually refers to overall glycemic variation including hyper- and hypoglycemia, GV is often also used to refer to postprandial glycemic excursion, especially in patients with T2DM. Thus, data interpretation should take into account different definitions and concepts of GV.

There are also various indices of GV, as summarized in Table 1. Standard deviation (SD) is most widely used to assess GV. Since SD is also positively associated with the mean value, the coefficient of variance (CV; SD divided by mean × 100 (%)) is also used to assess GV. Mean amplitude of glycemic excursions (MAGE) [10] and *M* value [11] are also frequently used. MAGE reflects relatively large glycemic excursions, but the measurement of MAGE may be subjective in terms of selection of large glycemic excursions. *M* value reflects glycemic variation from the baseline “ideal” value usually set at 90 to 120 mg/dL; thus, *M* value is also affected by the mean value. Continuous overlapping net glycemic action (CONGA) provides a precise measurement of within-day GV but requires CGM data for its calculation [12]. Mean of Daily Differences (MODD) [13] is used as an index of day-to-day GV. Although many other indices to assess GV have also been developed and each index reflects different aspects of GV, these indices of GV are largely correlated with each other and SD appears to remain the gold-standard index of GV [14,15].

Serum 1,5-anhydroglucitol (1,5-AG) is a marker of postprandial hyperglycemia. 1,5-AG is excreted into the urine but is normally fully reabsorbed by the renal tubules. When plasma glucose level rises (generally >180 mg/dL, the average renal threshold for glucose) and glucosuria occurs, serum 1,5-AG falls due to competitive inhibition of renal tubular reabsorption by glucose. Thus, a lower serum 1,5-AG level reflects higher glycemic excursion, which usually occurs after meals [16]. However, serum 1,5-AG level becomes too low to be used as a marker of glycemic excursion when marked hyperglycemia persists (e.g., HbA1c > 8%). Also, since treatment with acarbose interferes with and decreases serum 1,5-AG level independent of the plasma glucose level, serum 1,5-AG level may not accurately reflect glycemic excursion in patients treated with acarbose [17,18].

**Figure 2:**
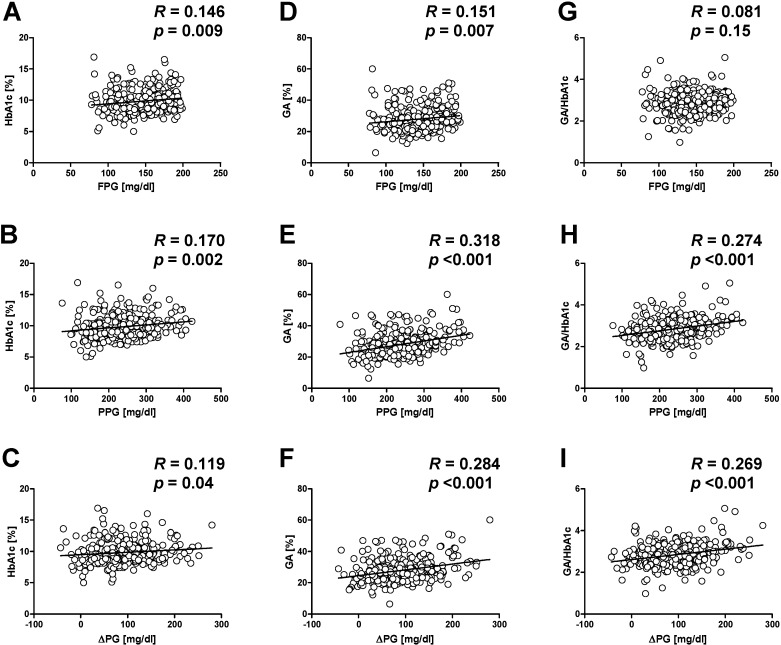
Correlations among HbA1c, glycated albumin (GA), GA/HbA1c ratio and other glycemic indices. HbA1c and GA were both significantly correlated with fasting plasma glucose (FPG) (**A**,**D**), postprandial plasma glucose (PPG) (**B**,**E**) and ∆PG (*i.e.*, PPG-FPG) (**C**,**F**), while GA was more strongly correlated with PPG and ∆PG than was HbA1c. There was a significant positive correlation between PPG (**H**) and ∆PG (**I**) and GA/HbA1c ratio, but no correlation between FPG and GA/HbA1c ratio (**G**). Reproduced with permission from the Japan Diabetes Society [21].

Glycated albumin (GA) is another marker of glycemic control. GA reflects average plasma glucose level over the past one to two weeks, while HbA1c reflects average plasma glucose level over the past one to two months [19]. Since albumin is more readily glycated compared with hemoglobin, GA also more sensitively reflects glycemic excursions and postprandial hyperglycemia compared with HbA1c [20]. We have reported that the ratio of GA to HbA1c was significantly correlated with postprandial plasma glucose level but not with fasting plasma glucose level, indicating that GA to HbA1c ratio reflects postprandial glucose excursion independently of fasting glucose level (Figure 2) [21].

**Table 1:** Indices of glycemic variability (GV).

Index	Description	Note
*Intra-day GV (needs SMBG or CGM)*		
SD		Commonly reported expression of GV.
CV	SD divided by mean	SD corrected for mean.
MAGE	Mean glucose value by summing absolute rises and falls of more than 1 SD	Smaller excursions of less than 1 SD are ignored. Determinant of glucose excursion could be subjective.
*M* value	Mean of logarithmic transformation of deviation from reference value	This formula puts greater emphasis on hypoglycemia than on hyperglycemia.
CONGA-*n*	SD of summed differences between current observation and observation *n* hours earlier	This calculation is more objective than MAGE. CGM data are needed for calculation.
*Between-day GV (needs SMBG or CGM)*		
MODD	Mean absolute value of differences between glucose values at the same time on two consecutive days	In daily practice, differences in mealtimes influence the value.
*Visit-to-visit GV*		
SD-FPG	SD of FPG over weeks to years	Reflects longer-term GV.
SD-HbA1c	SD of HbA1c over months to years	Reflects longer-term GV.
*Others*		
75 g OGTT	Assesses glycemic excursion after oral glucose load	Gold standard for diagnosis of glucose intolerance.
MTT	Reflects more physiological postprandial glycemic excursion	Needs standard meal for comparison.
1,5-AG	Value is lower in the presence of glucosuria	Reflects the presence of postprandial hyperglycemia.
GA	Reflects average glucose level over past 1 to 2 weeks	Reflects overall hyperglycemia and glycemic excursion.
Ratio of GA to HbA1c		Reflects glycemic excursion.

SD, standard deviation; CV, coefficient of variation; MAGE, mean amplitude of glycemic excursions; CONGA, continuous overall net glycemic action; MODD, mean of daily differences; SMBG, self-monitoring of blood glucose; CGM, continuous glucose monitoring; OGTT, oral glucose tolerance test; MTT, meal tolerance test; 1,5-AG, 1,5-anhydroglucitol; GA, glycated albumin.

## 3. Glycemic Variability and Vascular Events

In DCCT, it was reported that the incidence of diabetic complications was lower in subjects with intensive therapy using basal-bolus therapy or continuous subcutaneous insulin infusion (CSII) compared with those with conventional therapy, even with the same HbA1c levels [22]. This finding raised the hypothesis that a reduction in GV by intensive therapy might contribute to reduce the incidence of diabetic complications. However, the DCCT investigators recently reanalyzed the data and denied their previous conclusion [23]. Subsequent analyses of the DCCT data also found that there was no association between GV and micro- and macrovascular complications [24,25,26]. In DCCT, GV was assessed as SD and MAGE based on 7-point SMBG throughout the day.

On the other hand, epidemiological studies have consistently shown a significant association between postprandial hyperglycemia, but not fasting hyperglycemia, and incidence of cardiovascular events and all-cause mortality in the general population [27,28,29]. In these studies, postprandial hyperglycemia was assessed as the 120 min post-load plasma glucose level during 75 g oral glucose tolerance test (OGTT); however, it was also reported that lower 1,5-AG levels were associated with the development of CVD in a general population [30].

Esposito *et al.* reported that the postprandial incremental glucose peak that occurs mostly within 1 h after a meal was related to carotid intima-media thickness (IMT) in patients with T2DM [31]. In the San Luigi Gonzaga Diabetes Study, a 14-year follow-up of 505 patients with T2DM, HbA1c and 2 h postprandial blood glucose level after lunch were significantly associated with CVD events and mortality [32].

Recently, using CGM, Torimoto *et al.* have reported that reactive hyperemia index (RHI), an index of vascular endothelial function, was significantly correlated with SD and MAGE in patients with T2DM [33]. Notably, RHI was also correlated with the percentage of the time having hypoglycemia. The same group also reported a significant correlation between RHI and 1,5-AG [34]. Thus, these observational studies indicated an association between GV or postprandial glycemic excursion and the development of atherosclerosis in patients with T2DM. On the other hand, in the A1C-Derived Average Glucose (ADAG) study, Borg *et al.* reported that GV assessed by either SMBG or CGM did not significantly associate with known CVD risk factors in both T1DM and T2DM patients, while mean glucose values and HbA1c did [35]. In another study using CGM, Sartore *et al.* reported that there was a significant association between SD or CONGA-2 and the presence of retinopathy in patients with diabetes, although the correlation between GV and retinopathy was not significant in multivariate analysis [36].

In addition to daily GV, recent studies suggest that GV assessed over a longer period (*i.e.*, months to years) is also associated with vascular complications. A number of studies have reported that variability of fasting plasma glucose and HbA1c are associated with higher risk of development of retinopathy and nephropathy in patients with type 1 and type 2 diabetes [37,38,39,40,41,42]. Recently, Hirakawa *et al.* examined the association between visit-to-visit GV of FPG or HbA1c and vascular events in a cohort of the Action in Diabetes and Vascular Disease: the Preterax and Diamicron MR Controlled Evaluation (ADVANCE) trial, which consisted of 4399 patients with a median follow-up of 3 years [43]. They assessed visit-to-visit GV as the SD of five measurements of FPG or HbA1c during the first 3–24 months of the study. As a result, visit-to-visit GV of FPG and HbA1c were significantly associated with micro- and macrovascular events and mortality: highest *vs.* lowest tenth hazard ratio (95% confidence interval (CI)) 1.64 (1.05–2.55) for vascular events and 3.31 (1.57–6.98) for mortality. These results suggest that GV during a longer period is an important risk factor for vascular events and mortality, the impact of which may be equal to or even more than that of intraday GV.

## 4. Underlying Mechanisms: Oxidative Stress

### 4.1. Preclinical Studies

Reactive oxygen species (ROS) and oxidative stress are increased under hyperglycemia. It is hypothesized that oxidative stress and diabetic complications are linked through several pathways: (1) the polyol pathway; (2) hexosamine pathway; (3) protein kinase C activation; and (4) formation of advanced glycation end-products (AGEs) [44]. Studies have suggested that intermittent hyperglycemia rather than chronic hyperglycemia exaggerates the production of ROS.

In *in vitro* studies, it has been reported that intermittent high glucose levels (5 and 20 mmol/L every 24 h) stimulated ROS overproduction, which led to increased cellular apoptosis in human umbilical vein endothelial cells compared with that in a stable high glucose environment (20 mmol/L) [45,46,47].

*In vivo*, Horvath *et al.* examined the effect of “glycemic swings” on oxidative stress and endothelial function in streptozotocin-induced diabetic rats [48]. They treated the diabetic rats with either intermediate-insulin (insulin glargine) once daily to achieve steady normalization of blood glucose levels or long-acting insulin (ultralente insulin) once every other day to induce “glycemic swings” for 14 days. They found that diabetic rats with “glycemic swings” showed higher nitrotyrosine levels and endothelial dysfunction compared with rats with steady normalization of blood glucose. Endothelial dysfunction in rats with “glycemic swings” was even more pronounced than that in untreated diabetic rats.

### 4.2. Clinical Studies

In humans, Ceriello *et al.* examined the effect of oscillating glucose on oxidative stress and endothelial function in healthy subjects and patients with type 2 diabetes [49]. They conducted a euinsulinemic hyperglycemic clamp to compare three different glycemic profiles over 24 h: (1) 10 mmol/L constant; (2) 15 mmol/L constant and (3) 5 and 15 mmol/L every 6 h (oscillating glucose). As a result, oscillating glucose resulted in further deterioration of endothelial dysfunction and oxidative stress assessed by plasma 3-nitrotyrosine and 24 h urinary excretion rate of 8-iso-prostaglandin F2α (8-iso-PGF2α) compared with either continuous 10 or 15 mmol/L glucose. These changes with hyperglycemia were reversed by concomitant vitamin C infusion, suggesting that increased oxidative stress was the cause of endothelial dysfunction.

In 2006, Monnier *et al.* reported a strong positive correlation between MAGE assessed by CGM and an oxidative stress marker, 24 h urinary excretion rate of 8-iso-PGF2α, in 21 patients with T2DM (*r* = 0.86, *p* < 0.001) [50]. This study was the first to use CGM to assess the relationship between GV and oxidative stress, and underpinned the important role of GV in increased oxidative stress in patients with diabetes. However, subsequent studies have shown conflicting results. Wentholt *et al.* examined the correlation between GV and oxidative stress in 25 patients with T1DM [51]. They assessed GV with MAGE, MODD and CONGA-1 calculated from CGM data and oxidative stress with 24 h urinary excretion rate of 8-iso-PGF2α. Although higher levels of 8-iso-PGF2α were observed in patients with T1DM compared with healthy controls, there was no correlation between GV and oxidative stress in these patients. Siegelaar *et al.* also reported no significant association between GV and urinary 8-iso-PGF2α excretion in patients with T2DM well-controlled with oral antidiabetic drugs (OAD) [52].

On the other hand, Di Flaviani *et al.* reported that there was a significant association between CONGA-2 and urinary 8-iso-PGF2α excretion in 26 patients with T2DM treated with diet and/or metformin [53]. In this study, a significant association between CONGA-2 and left ventricular mass index (LVMI) was also reported. In addition to the differences in patients’ characteristics and medications, different methods of oxidative stress measurement (e.g., ELISA *vs.* tandem mass spectrometry for 8-iso-PGF2α measurement) may have caused these conflicting results among studies [54].

## 5. Other Mechanisms

### 5.1. Dyslipidemia

In addition to hyperglycemia-induced oxidative stress, postprandial dyslipidemia, mainly triglyceridemia, may also contribute to vascular damage and CVD [55].

### 5.2. Glycated Albumin

GA has not only been shown to be a marker of GV, but GA itself has also been postulated to promote atherosclerosis [56]. Since glycation of albumin impairs the antioxidant activities of albumin, GA may contribute to increased oxidative stress in patients with diabetes [57]. GA or GA to HbA1c ratio, but not HbA1c, was associated with carotid IMT or plaque, and severity of coronary atherosclerosis [58,59,60,61].

Recently, the predictive value of GA for vascular complications has been evaluated in two large prospective cohorts of DCCT and the Atherosclerosis Risk in Communities (ARIC) study [62,63]. As a result, GA has been shown to have similar predictive value to HbA1c for the development of microvascular complications in patients with T1DM and T2DM, and a combination of both improved the predictive value; however, the association between GA and the development of CVD was not confirmed in DCCT.

### 5.3. Hypoglycemia

Hypoglycemia is another possible link between GV and poorer CVD outcomes. It has been reported that greater GV predicted more frequent hypoglycemic events, including severe hypoglycemia requiring assistance from a third person, in patients with T1DM [64,65,66]. In the ACCORD study, all-cause mortality was higher in the intensive glucose control group compared with the conventional treatment group [5]. The incidence of all and severe hypoglycemia was significantly higher in the intensive glucose control group, although the investigators concluded that hypoglycemia was not a cause of increased death in the intensive therapy group [67].

Nonetheless, severe hypoglycemia has been shown to predict all-cause mortality in patients with T2DM [67,68,69]. Although whether hypoglycemia is causative of death or a coincidental marker of illness has not been fully elucidated, hypoglycemia could induce the onset of CVD through induction of (1) inflammation; (2) blood coagulation abnormality; (3) sympathoadrenal response and (4) endothelial dysfunction [70]. Recent studies suggest that hypoglycemia is associated with impaired cardiovascular autonomic function and an increased risk of arrhythmia, especially nocturnal arrhythmia, which may lead to a sudden death, so called “dead-in-bed” syndrome [71,72,73,74,75].

Ceriello *et al.* recently reported the effect of hyperglycemia after hypoglycemia under a glucose clamp in healthy subjects and subjects with T1DM. Hypoglycemia induced for 2 h increased oxidative stress and worsened endothelial function, which was further worsened by hyperglycemia after hypoglycemia [76].

## 6. Clinical Implications

### 6.1. Effects of Treatment of Glycemic Variability on Oxidative Stress and Cardiovascular Outcomes

As described above, *in vitro* and *in vivo* animal studies and observational and experimental human studies indicate that oxidative stress is a plausible link between GV and CVD. However, the results of intervention studies are more conflicting.

The Hyperglycemia and its Effect after Acute myocardial infarction on cardiovascular outcomes in patients with Type 2 Diabetes mellitus (HEART2D) trial is to date the only study to directly compare the effects of postprandial *vs.* fasting glycemic control on CVD outcome [77]. A total of 1115 patients with T2DM who had had an acute myocardial infarction within 21 days were assigned to either a prandial strategy group or basal strategy group. The patients in the prandial strategy group were treated with three pre-meal insulin lispro, a rapid-acting insulin analog, injections with a target of 2 h postprandial blood glucose <7.5 mmol/L (135 mg/dL) and those in the basal strategy group were treated with neutral protamine Hagedorn (NPH) insulin twice daily or insulin glargine, a long-acting insulin analog, once daily with a target of fasting/pre-meal blood glucose <6.7 mmol/L (121 mg/dL). The trial was stopped because of lack of efficacy, with a mean follow-up period of 963 days. During the study, HbA1c was similarly reduced in both groups, and the prandial strategy group showed a lower daily mean postprandial blood glucose compared with the basal strategy group. There was no significant difference in incidence of first CVD event between the groups (hazard ratio 0.98, 95% CI 0.8–1.21). The incidence of all and of severe hypoglycemia were similar between the groups. However, post-hoc subgroup analysis showed that in subjects aged >65.7 years (*n* = 399), fewer CVD events were observed in the prandial strategy group compared with the basal strategy group (hazard ratio 0.69, 95% CI 0.49–0.96, *p* = 0.03) [78].

The results of this study were criticized in view of the fact that the difference in postprandial blood glucose level between the groups was less than expected (0.8 mmol/L (14.4 mg/dL) compared with the expected goal of 2.5 mmol/L (45 mg/dL)). Improvement of the management of other coronary risk factors might also have affected the results. Nonetheless, it should be acknowledged that this study was designed to compare two different insulin regimens rather than to clarify the role of GV in CVD outcome.

It has been reported that a similar reduction of oxidative stress was obtained by nine days of treatment with either inhaled mealtime insulin or basal insulin in patients with T2DM [79]. Moreover, Monnier *et al.* reported that urinary 8-iso-PGF2α level was decreased by insulin treatment but not OAD treatment in patients with T2DM [80], suggesting that insulin therapy itself may induce an anti-oxidative effect independently of hyperglycemia or GV, and this anti-oxidative effect of insulin might have affected the results of the HEART2D trial.

α-Glucosidase inhibitors (AGI) slow absorption of glucose from the intestine, resulting in suppression of postprandial glucose excursion. In the Study to Prevent Non-Insulin-Dependent Diabetes Mellitus (STOP-NIDDM) study, treatment with acarbose reduced the incidence of T2DM in patients with impaired glucose tolerance (IGT) [81]. In this study, a reduction in CVD in patients treated with acarbose was also reported [82]. A reduction in CVD by treatment with acarbose was also observed in patients with T2DM [83], suggesting the importance of reduction in postprandial excursion to prevent CVD in patients with IGT and T2DM.

Glinides are rapid-acting insulin secretagogues, thereby reducing postprandial glucose excursion similarly to AGIs. Esposito *et al.* reported that treatment with repaglinide reduced postprandial hyperglycemia compared with the effect of glyburide, a sulfonylurea, and more patients treated with repaglinide showed regression of carotid IMT compared with those treated with glyburide [84]. Mita *et al.* also reported that treatment with nateglinide resulted in regression of carotid IMT compared with that in untreated patients with T2DM [85].

On the other hand, in the Nateglinide and Valsartan in Impaired Glucose Tolerance Outcomes Research (NAVIGATOR) trial, treatment with nateglinide did not reduce the incidence of either T2DM or CVD [86]. Differences in patients’ characteristics and an insufficient dose of nateglinide used in the study might have affected these results. However, the different results between the STOP-NIDDM and NAVIGATOR trials may be associated with the different mechanisms of action of the two drugs. As AGIs slow glucose absorption, postprandial insulin secretion is reduced. On the other hand, glinides suppress postprandial glucose excursion through increasing early phase insulin secretion after meal ingestion. Recently, Sawada *et al.* have reported a comparison of the effects on oxidative stress and endothelial function between a glinide and an AGI [87]. In this study, a total of 104 patients with T2DM were randomly assigned to treatment with miglitol or nateglinide. After 4 months of treatment, despite similar improvement of HbA1c and 1,5-AG in both groups, a reduction in oxidative stress assessed by diacron reactive oxygen metabolites (d-ROMs) and improvement of percent flow-mediated dilatation (%FMD) were observed only in the patients treated with miglitol, accompanied by improvement of insulin resistance and lipid profile, suggesting that treatment of postprandial glucose excursion without stimulating insulin secretion may be preferable to ameliorate endothelial dysfunction through a reduction in oxidative stress. Whether endogenous and exogenous insulin have different effects on oxidative stress remains unknown.

We have also recently reported the effects of mitiglinide on GV and oxidative stress markers in patients with T2DM [88]. Treatment with mitiglinide for 4 months significantly improved 1,5-AG and daily GV assessed by 7-point SMBG, but there was no change in plasma oxidized low-density lipoprotein (oxLDL), plasma pentosidine, urinary excretion of 8-hydroxydeoxy guanosine (8-OHdG) or 8-iso-PGF2α after treatment. On the other hand, Wang *et al.* have reported that treatment with nateglinide for 4 weeks significantly improved insulin resistance, oxidative stress (nitric oxide, malondialdehyde and superoxide dismutase) and endothelial dysfunction in patients with newly diagnosed T2DM [89].

Dipeptidyl peptidase 4 (DPP-4) inhibitors increase insulin secretion and suppress glucagon secretion in a glucose-dependent manner through enhancement of the effect of endogenous incretin, thereby improving postprandial glycemic excursion without increasing the risk of hypoglycemia. Thus, treatment with DPP-4 inhibitors is expected to improve GV [90,91]. Rizzo *et al.* compared the effect on GV and oxidative stress between two different DPP-4 inhibitors, sitagliptin and vildagliptin, in patients with T2DM [92]. After 12 weeks of treatment, HbA1c was similarly improved in both groups; however, MAGE was significantly lower in patients treated with vildagliptin compared with those treated with sitagliptin. Oxidative stress assessed by nitrotyrosine and inflammatory markers (interleukin (IL)-6 and IL-18) was significantly lower in the vildagliptin group, and there was a significant correlation between nitrotyrosine and change in MAGE but not HbA1c. The same researchers also reported that weight loss after bariatric surgery resulted in a reduction in MAGE and plasma nitrotyrosine levels accompanied by increased glucagon-like peptide 1 (GLP-1) level in patients with T2DM, although no change in MAGE and plasma nitrotyrosine levels was observed after weight loss by dieting [93].

These favorable changes in GV without an excess risk of hypoglycemia by treatment with DPP-4 inhibitors are expected to result in improvement of CVD outcome. However, recently two randomized controlled trials failed to show a beneficial effect of DPP-4 inhibitors on CVD outcome [94,95].

GLP-1 receptor agonists (GLP-1RA) also lead to a supraphysiological activation of GLP-1 receptor and improve GV. Ceriello *et al.* reported that GLP-1 infusion improved oxidative stress assessed by plasma nitrotyrosine and 8-iso-PGF2α and endothelial dysfunction induced by hyper- and hypoglycemia in patients with T1DM [96]. This effect was further augmented by concomitant vitamin C infusion [97]. They also reported additive beneficial effects of a combination of GLP-1 and insulin on hyperglycemia-induced oxidative stress and endothelial dysfunction in patients with T2DM [98]. However, this effect of GLP-1 was independent of GV since the same plasma glucose level was maintained during the study. Kelly *et al.* reported that there was no significant change in oxidative stress (oxLDL) and endothelial function assessed by RHI between baseline and after 3 months of treatment with either exenatide or metformin in patients with prediabetes [99].

Thus, to date, the causative role of GV in oxidative stress and CVD in patients with diabetes remains controversial. Finally, it was reported that antioxidant vitamin supplementation did not improve CVD outcome and all-cause mortality in the general population and patients with diabetes [100,101].

### 6.2. Factors Associated with Glycemic Variability

For the management of GV, it is important to clarify factors associated with GV in patients with diabetes. Factors that associate with GV are summarized in Table 2.

**Table 2:** Factors associated with greater glycemic variability.

Reduced β cell function
Older age
Liver failure
Renal impairment
Reduced lean mass
Autonomic neuropathy
Anti-diabetic medication
Polypharmacy
Cognitive impairment/dementia
Poor compliance with treatment
Intake of food with higher glycemic index and/or glycemic load
Amount of vegetables/fiber intake
Irregular timing of meals
Physical inactivity

**Figure 3:**
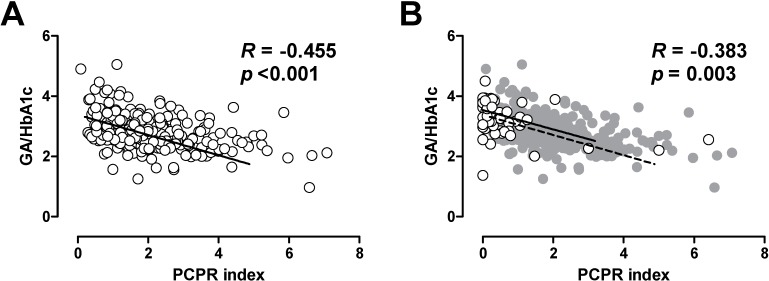
Correlation between postprandial C-peptide index (PCPRI) and glycated albumin (GA) to HbA1c ratio in patients with type 2 diabetes (**A**) and type 1 diabetes (**B**). (**B**) The data of patients with type 1 diabetes are superimposed on the data of those with type 2 diabetes (gray circles and dotted line). Reproduced with permission from the Japan Diabetes Society [21].

A defect of beta cell function is a hallmark of both type 1 and type 2 diabetes [102,103]. An inverse relationship between residual C-peptide level and GV has been reported in patients with T1DM [104,105,106]. We and others reported that an inverse relationship between beta cell function and GA to HbA1c ratio was observed in patients with T2DM [21,107,108,109,110] (Figure 3A), suggesting that less beta cell function is associated with greater postprandial glycemic excursion or GV in patients with T2DM. Interestingly, the relationship between beta cell function assessed by serum C-peptide and GA to HbA1c ratio was comparable between patients with type 1 and type 2 diabetes [21] (Figure 3B), suggesting that the relationship between serum C-peptide and GV is independent of the type of diabetes. Kramer *et al.* recently reported the effects of intensive insulin therapy on GV and beta cell function in 61 patients with early-stage T2DM [111]. Intensive insulin therapy for 4 weeks reduced GV assessed by 6-point SMBG, and the reduction in GV was significantly associated with improvement of beta cell function.

**Figure 4:**
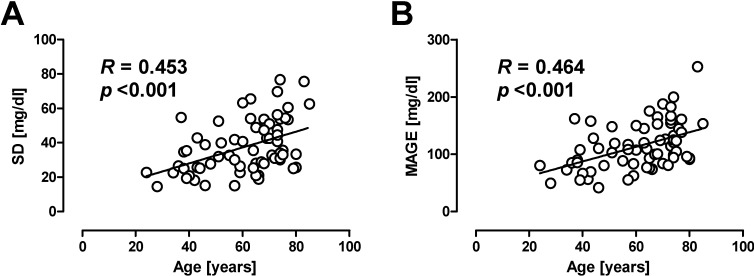
Correlation between age and (**A**) standard deviation (SD) or (**B**) mean amplitude of glycemic excursions (MAGE) assessed by CGM in patients with T2DM. Reproduced with permission from the Japan Diabetes Society [116].

Age is another factor associated with GV. Munshi *et al.* reported that the proportion of postprandial hyperglycemia in total hyperglycemia was greater in older (≥65 years) patients with T2DM compared with younger (<65 years) patients [112]. We have also reported that SD and MAGE assessed by CGM were significantly associated with age in patients with T2DM (Figure 4). The progressive decline in beta cell function with duration of diabetes, impairment of multiple organs such as liver and kidney, reduction in lean mass, and autonomic neuropathy may be attributable to the relationship between age and GV. In addition, polypharmacy and cognitive impairment/dementia, resulting in poor compliance with medication, may also contribute to greater GV in the elderly. A significant inverse association between MAGE and cognitive function has been reported in older patients with T2DM [113]. Therefore, the increased risk of hypoglycemia in older patients with T2DM [114,115] is at least in part due to greater GV in this population.

### 6.3. Importance of Preventing Hypoglycemia

**Figure 5:**
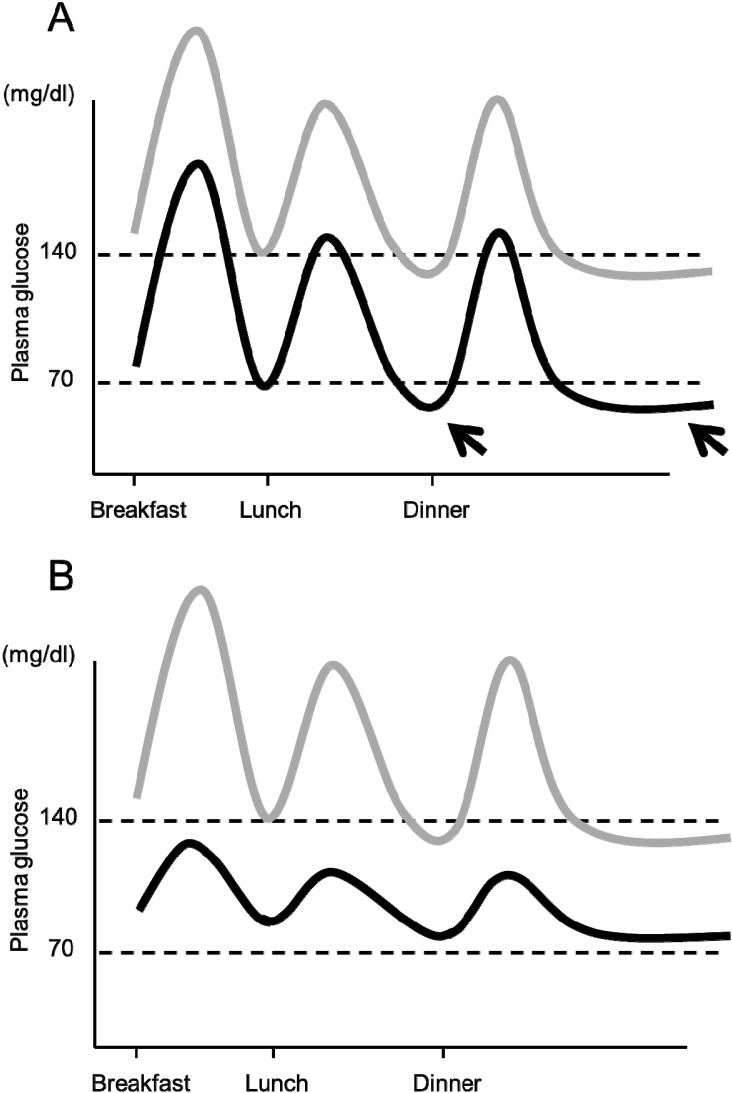
Importance of controlling postprandial glycemic excursion for prevention of hypoglycemia. (**A**) If mean plasma glucose level is lowered without controlling postprandial glycemic excursion (gray line → black line), the risk of pre-meal and nocturnal hypoglycemia increases (arrows); (**B**) Lowering the mean glucose level with correction of postprandial glycemic excursion (gray line → black line) results in a low risk of hypoglycemia. Note that the mean plasma glucose level is similar in both cases, indicating similar HbA1c in both cases.

Although the relationship between GV and progression of atherosclerosis remains to be established, importantly, it has been reported that GV is associated with hypoglycemia, especially severe hypoglycemia [64,65,66]. Severe hypoglycemia can not only directly cause death, but also predict higher risk of mortality [67,68,69]. Hypoglycemia may also increase a risk of CVD or severe arrhythmia, as described above. Thus, clinically, minimizing GV is important not only for prevention of CVD but also for prevention of hypoglycemia in the management of diabetes (Figure 5).

### 6.4. Treatment Strategy to Minimize Glycemic Variability

As described above, beta cell function is one of the major factors associated with GV. Moreover, beta cell function progressively deteriorates during the course of the disease [117,118,119]. Therefore, to appropriately manage GV, it is important to preserve and recover beta cell function.

**Figure 6:**
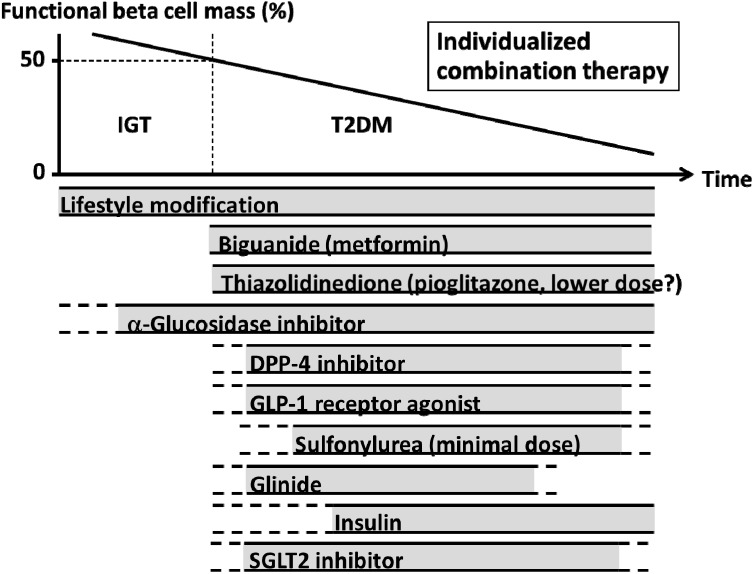
Proposed concept of treatment strategy for type 2 diabetes (T2DM) in relation to functional beta cell mass. An α-glucosidase inhibitor is partly approved for use in patients with impaired glucose tolerance (IGT) in Japan. Medications not approved or marketed in Japan are not included in the figure. Since currently no single therapy or agent can cure or even manage T2DM, an effective and individualized combination of current medications in addition to lifestyle modification aiming at reduction in beta cell workload is important to preserve or recover beta cell function, which may lead to a reduction in risk of severe hypoglycemia.

To date, a reduction in beta cell workload appears the most effective approach to preserve or recover beta cell function [103,118]. Thus, use of biguanides and/or thiazolidinediones is recommended to preserve beta cell function without increasing the risk of hypoglycemia, if it is not possible to achieve the glycemic goal with lifestyle modification (Figure 6).

To specifically reduce postprandial glycemic excursion, AGI and glinides are recommended. Since sulfonylureas (SUs) do not effectively reduce GV, and increase the risk of hypoglycemia [120,121], their use may be somewhat limited, especially in older patients. In this case, glinides are recommended as a substitute for SUs. In addition to general lifestyle modification, selection of food with lower glycemic index, increasing dietary fiber and implementing a brisk walk after meals are recommended to reduce postprandial glycemic excursion [122,123].

As incretin drugs, *i.e.*, DPP-4 inhibitors and GLP-1RAs, act in a glucose-dependent manner, the use of these drugs also improves GV [121,124]. Short-acting GLP-1RAs seem to more effectively reduce postprandial glycemic excursion compared with long-acting GLP-1RAs, through inhibiting gastric emptying without enhancing insulin secretion [125]. Sodium glucose cotransporter 2 (SGLT2) inhibitors, which have recently been approved in several countries, also reduce GV without enhancing insulin secretion [126,127].

Insulin therapy is eventually needed in most patients with T2DM. Rapid-acting insulin analogs such as insulin aspart, insulin lispro and insulin gluligine are used to control postprandial hyperglycemia, although the use of prandial insulin results in a higher risk of hypoglycemia and weight gain compared with the use of basal insulin [128].

## 7. Conclusions

This review summarizes the current knowledge on the associations among GV, oxidative stress and CVD, especially focusing on clinical evidence, and discusses their clinical implications in the management of GV.

*In vitro* and *in vivo* animal studies indicate that GV, compared with chronic hyperglycemia, promotes excess oxidative stress and worsens cellular and vascular damage. In experimental settings, several studies have also confirmed these findings in humans.

However, the effect of GV on oxidative stress in clinical settings remains controversial, and to date no solid evidence exists. Few studies have demonstrated the effect of daily GV on CVD outcomes, although visit-to-visit GV, a longer term index, may predict worse CVD outcome in patients with diabetes. In contrast, the association between postprandial hyperglycemia and CVD has been consistently reported in the general population. Different definitions or concepts (e.g., glycemic variability *vs.* postprandial glucose excursions) of GV further complicate the interpretation of the data, although they are correlated with each other and not able to be completely separated. In addition, the conflicting findings among the numerous studies may be derived from differences in study design, intervention period, baseline patient characteristics, use of different medications and use of different oxidative stress markers. Although plasma or urinary 8-iso-PGF2α is widely used for assessment of oxidative stress, the two different methods of measurement (ELISA and tandem mass spectrometry) may affect the findings.

These conflicting results may also suggest that the effect of daily GV on oxidative stress or CVD is, if anything, minimal to modest in patients with diabetes. It has been shown that multifactorial intervention for coronary risk factors effectively reduces the incidence of CVD and all-cause mortality [129]. Antihypertensives and statins have also been reported to reduce oxidative stress [130,131,132,133]. Thus, to improve CVD outcomes and mortality in patients with diabetes, a multifactorial approach appropriately managing each coronary risk factor is necessary.

Nonetheless, the association between GV and hypoglycemia is clinically important and thus GV should be minimized to prevent hypoglycemia in clinical settings. Since GV becomes greater when beta cell function decreases, treatment of patients with diabetes should focus on preservation or recovery of beta cell function. Also, since GV becomes greater with age, older patients need to be treated with special caution in regard to hypoglycemia.

In conclusion, further research including prospective trials to explore the mechanisms linking GV and CVD is warranted. Although the relationship among GV, oxidative stress and CVD has not been established, continuous efforts to minimize GV will prevent hypoglycemia and improve QOL in patients with diabetes, and hopefully lead to the improvement of CVD outcome in patients with and without diabetes.
